# Intelligence and global health: assessing the role of open source and social media intelligence analysis in infectious disease outbreaks

**DOI:** 10.1007/s10389-018-0899-3

**Published:** 2018-02-10

**Authors:** Rose Bernard, G. Bowsher, C. Milner, P. Boyle, P. Patel, R. Sullivan

**Affiliations:** 1 Conflict and Health Research Group, King’s College London, London, UK; 2 King’s Centre for Global Health, Suite 2.13 Weston Education Centre, Cutcombe Road, London, SE5 9RJ UK; 3 International Prevention Research Institute, Lyon, France; 4 Department of War Studies, King’s College London, London, UK

**Keywords:** Epidemics, Intelligence, Global health, OSINT, SIGINT

## Abstract

**Purpose:**

Open Source Intelligence (OSINT) and Signals Intelligence (SIGINT) from the clandestine intelligence sector are being increasingly employed in infectious disease outbreaks. The purpose of this article is to explore how such tools might be employed in the detection, reporting, and control of outbreaks designated as a ‘threat’ by the global community. It is also intended to analyse previous use of such tools during the Ebola and SARS epidemics and to discuss key questions regarding the ethics and legality of initiatives that further blur the military and humanitarian spaces.

**Methods:**

We undertake qualitative analysis of current discussions on OSINT and SIGINT and their intersection with global health. We also review current literature and describe the debates. We built on quantitative and qualitative research done into current health collection capabilities.

**Results:**

This article presents an argument for the use of OSINT in the detection of infectious disease outbreaks and how this might occur.

**Conclusion:**

We conclude that there is a place for OSINT and SIGINT in the detection and reporting of outbreaks. However, such tools are not sufficient on their own and must be corroborated for the intelligence to be relevant and actionable. Finally, we conclude that further discussion on key ethical issues needs to take place before such research can continue. In particular, this involves questions of jurisdiction, data ownership, and ethical considerations.

## Introduction

Infectious disease outbreaks—SARS, MERS, Ebola—have increasingly come to be designated as potent threats to the international community under the escalating global health security agenda (Smith 2017). Interconnected trade, low-cost air travel and increasing population mobility continue to make infectious disease outbreaks an issue of significant concern to the global community (Wenham 2016).

Here we explore the extent to which tools and concepts of national intelligence services might aid the detection and control of infectious disease outbreaks. We explore the potential overlap between techniques used by national intelligence services and those used for infectious disease detection, particularly with regard to analysis of Open Source Intelligence, and we consider the ethical implications in blending civilian and military methodology in the context of global health.

## Open source intelligence (OSINT)

Open Source Intelligence is defined by the US Department of Defence (DoD) as ‘produced from publicly available information…’ (National Defence Authorisation Act for Fiscal Year 2006). It is a wide definition that encompasses any source openly available and includes media sources that can be accessed instantly, with potential use in outbreak alerts. OSINT is currently in use globally in certain surveillance systems and forms the foundation of many public intelligence capabilities. The public health community has begun to recognise the scope of these services, and many projects currently exist that explore how OSINT might help identify and monitor diseases that constitute a Public Health Emergency of International Concern (PHEIC) (World Health Organisation 2008).

The World Health Organisation (WHO) operates a programme to assist in the collection and assessment of OSINT in disease intelligence. The Global Public Health Intelligence Network (GPHIN) is a crucial part of a larger platform developed by the WHO named the Hazard Detection and Risk Assessment System (HDRAS), which uses web-based epidemic intelligence tools and collects information from Medisys, GPHIN, Healthmap, and Promed-mail, amongst others. GPHIN is a semi-automated early warning system that continuously scans global media sources in nine languages, searching for key words, phrases, and any potential signs of disease outbreaks (Mawadeku 2007). It has the capacity to generate between 2000 and 4000 daily reports and produce alerts automatically.

OSINT tools applied to health surveillance, such as GPHIN, are able to automatically collect and collate data, thereby referencing much larger quantities of information. Algorithms are used to filter relevant reports. GPHIN, ProMED, and HealthMap have been able to provide alerts on some of the most serious outbreaks since the turn of the century. By evaluating content from Chinese media and low-level chatter, (Lerbinger 2012), ProMED provided the first English language alert of SARS and even ‘prompted’ subsequent confirmation by the Chinese Government (Milinovich et al. 2015). Some signs for the recent Ebola outbreak were detected by HealthMap before any official announcement because of its ability to scan news stories in the local language (Milinovich et al. 2015).

Public health surveillance is also exploring the exploitation of Social Media Intelligence (SOCMINT). SOCMINT uses the growing proliferation of social media and web forums globally to provide contemporaneous information on a specified topic or theme. The geographic availability of SOCMINT is less limited than that of OSINT: while traditional internet coverage rates in many areas of the world are low, mobile data coverage in these regions is growing. The most popular services accessed through mobile data are often social media sites. In these instances, Facebook is often used for news and has potential to deliver more raw information than traditional publishing sources.

Digital Disease Detection (DDD, also known as digital disease surveillance) has already been used in many ways in the field of epidemiology—in the creation of the public health systems mentioned above, but also in time series analysis for investigation of the period preceding the outbreak, and for assessing public sentiment regarding the perceived impact of a disease and the proportionality of government or health service response (Salathé et al. 2013). Many initiatives have attempted to demonstrate the potential of search-term surveillance. This technique exploits data on searches made by members of the public to compile trend data based on keywords. Most significantly, in 2008 Google launched Google Flu Trends (GFT), an attempt to aggregate data based on the volume of public searches for keywords related to influenza and to use this to predict when an outbreak might occur (Cook et al. 2011). The principle behind this was that when people are ill with an influenza-like illness (ILI), they search for keywords relating to an influenza-like illness. However, in a review in 2014, researchers identified that GFT had a trend of results corresponding with ILINet—the US surveillance programme for ILI—for 2 to 3 years before deviating in a spectacular way, for example missing the flu season in 2013 by 140% (Lazer et al. 2013).

The failure of GFT highlights one of the greatest limitations in the use of OSINT—that any information must be assessed before it can be turned into intelligence. Although we are now able to exploit algorithms to extract statistically important information from unstructured data sets, such information must still be assessed by analysts and researchers. A large data set is no use unless we are querying it properly, and this holds true for any repository of information regarding public health. For example, in a crisis situation Non-Governmental Organisations (NGOs) do not necessarily need full satellite images of a disaster zone, and to provide them with such would create more work going through them. What they do need is the specific information that these images can show, such as damage to infrastructure and population movements (Clunan 2006). OSINT used on its own is therefore not sufficient; it must be corroborated from other sources and agents (Hulnick 2002). GPHIN and similar systems will suffer from the distortion created by the heuristic availability. Additionally, just because multiple news sources publish or report on an event does not verify either its accuracy or its authenticity—OSINT must be verified against other information (Pallaris 2008). This is especially true for infectious diseases that require laboratory verification (World Health Organisation 2014). As the ‘quality of information is [not] controlled… rumours [may] prove to be unsubstantiated’ (Grein 2000). GFT’s initial algorithm had a high rate of failure due to overfitting of unrelated search terms. A review of biosurveillance systems found that GPHIN combined with human moderation improves detection rates by 53%, reinforcing the importance of human factors despite technological advances (Barboza et al. 2014). Such systems must still be used in conjunction with physical verification teams to add real value, since limitations remain in the rapid reporting of outbreaks as they unfold.

Such are the limitations of big data. Similarly, OSINT utilised in this manner has a number of limitations. First, pertinent pieces of information can often be lost amongst the background noise of the internet because of the sheer quantity of data available. Indeed, one of the greatest issues with OSINT is that there can be so much data that deriving analytics becomes difficult (Hulnick 2002). Indeed, GPHIN was unable to verify over 30% of its total reports in 2002, despite generating the highest number of potential alerts that year in comparison to other reporting methods (Birnbaum 2013).

While the exploitation of new sources of intelligence is critical in the adaptation of public health surveillance, we must consider the techniques that intelligence services use to make information relevant and actionable. The most significant change is the move from static to active data and mapping. Such techniques have been used by the military since generals could push representative carved pieces across maps, but active open source intelligence allows for a near contemporary representation that can be used in operational planning. Both the advantages and disadvantages of such techniques were demonstrated in the use of crisis mapping during the earthquake in Haiti in 2010 (Zook et al. 2010). While the map acted as a way of mapping incidents and needs, including medical needs, and translating these to aid workers in a way that allowed them to specifically address the crisis, the variety of sources, the incompatibility of data platforms, and the sheer amount of information overwhelmed aid workers, hindering relief organisation efforts as well as helping (Gao et al. 2011). More recent iterations of crisis mappings have improved upon this: by the time the Standby Volunteer Task Force (SBTF) were deployed to provide crisis mapping assistance in the Libyan Conflict in February 2011, clearer intelligence pathways had been created in advance and included open and closed versions of the map to protect sources (United Nations Office for the Coordination of Humanitarian Affairs 2011).

Such techniques have not yet been replicated in the case of pandemics or PHEICs. HealthMap comes the closest, but what differentiates HealthMap from current crisis mapping is that crisis mapping is activated once a crisis has been declared, and volunteers have a defined set of criteria to monitor. This makes it much easier to cut through the noise of information. HealthMap captures information globally, meaning that it remains difficult to separate events that have the potential to become critical from more endemic cases. Sharper data collection, an intelligence cycle, and established intelligence pathways are all tools that must be explored with regard to the use of contemporaneous crisis mapping in infectious disease. Given that health and humanitarian crises are increasingly overlapping, the use of the framework created by the SBTF for crisis mapping during humanitarian events is something that merits further exploration.

However, in creating such maps, and particularly in the exploitation of search engine data, we must consider that it is difficult to implement OSINT in low-middle income countries [LMICs]. These resource-poor settings, particularly fragile and post-conflict settings, are often geographically the most likely sources of outbreaks designated as health security threats; yet, their communications infrastructure and digital presence are often limited (Eysenbach 2003). OSINT tools like GPHIN and HealthMap have a ‘clear bias towards countries with higher numbers of media outlets…and greater availability of electronic communication infrastructure’ (Brownstein et al. 2008). A technical officer at the WHO confirmed this as the biggest limitation to using OSINT in disease surveillance.^1^

Finally, the ethics around using social media intelligence remain contested. Particularly in the case of infectious disease outbreaks that have previously been used as proxy methods to victimise or discriminate against a portion of a population, there is a high risk of contributing to such discrimination if the sources are made public. This was demonstrated when internet users identified the wrong suspects after the Boston Marathon bombings in 2013, which led to an innocent student being identified and victimised. Furthermore, social media is continually redefining our concepts of personal and private. Although information shared on social networking sites such as FaceBook may be public, we still expect a contextual degree of privacy and it is as yet unclear under which circumstances such expectations may be set aside in the name of public health (Bradwell 2010). Such concerns could be alleviated by asking for consent, but again we risk an availability bias in that those with a digital presence are most likely to come from the countries with lower likelihoods of infectious disease outbreaks designated as health security threats. Prior to any systematic exploitation of such data we therefore need to consider carefully a framework under which we can do so (Omand et al. 2012).

## Signals intelligence (SIGINT)

The intelligence technique that is least commonly used in infectious disease surveillance is Signals Intelligence (SIGINT). Signals Intelligence is the collection of communication data, often through telephone and email interception. It can also encompass internet metadata and location data. Due to the sensitive nature of SIGINT, legal collection is most often undertaken by national intelligence agencies and law enforcement, who may either target specific individuals or collect data in bulk, where it is appropriate to do so. Programmes such as the US National Security Agency’s PRISM and X-Keyscore, or the British Government Communications Headquarters GCHQ’s Tempora, are now known to be able to collect communications data on a mass scale through the interception of internet servers, satellites, fibre-optic cables, telephone systems and personal computers (Greenwald 2015).

Various proposals have been made for the employment of SIGINT as a tool in outbreak surveillance, including the collection of mass communication data. Algorithms could be used to identify alerts of potential disease outbreaks by applying search conditions to pre-existing data sets or data collection parameters. Expanding extant collection programmes to include targeted global health priorities would then provide alerts for human operators to analyse and validate—adding to Early Warning and Response and detecting diseases early. Other proposals suggest that SIGINT may be of use in contact tracing—identification and diagnosis of people who may have come into contact with an infected person.

However, there are many problems relating to the use of SIGINT in infectious disease surveillance. First and foremost are the ethical and legal issues, particularly in a post-Snowden era where bulk data collection is a politically sensitive topic. Greenwald believes privacy is the biggest issue with SIGINT, as millions of ordinary people are subjected to severe breaches of privacy through their communications being intercepted with little accountability (Greenwald 2015). Other such studies have raised similar concerns (Freifeld et al. 2010). Using national intelligence capabilities in this context would be unpalatable for many, and it is unlikely that surveillance would meet the thresholds for necessity and proportionality that would balance out a high risk of collateral intrusion. In the contexts that SIGINT could be practically deployed, the value added to disease surveillance would be minimal since high-income countries already maintain strong disease surveillance systems within their national boundaries. Further, privacy breaches of this kind have unknown effects as they play out in health and human domains in complex geo-political settings. There are also further questions about what would happen to the data after it has been collected and whether it could be used by host governments to discriminate against groups within a population.

The use of SIGINT is governed by national legislation, and any additional collection would be constrained by this legal framework. These surveillance programmes are highly contentious: GCHQ has been challenged in the European Court of Human Rights (ECHR) over its collection under the Data Retention and Investigatory Powers Act 2014, and the NSA has faced significant criticisms in the aftermath of Snowden. Widespread deployment of SIGINT therefore necessitates the implementation of a legal process allowing for oversight by a relevant authority. Governance of this kind within the field of global health might be expected to take place within the WHO or other organisations of the UN system, however given the highly sensitive nature of the associated technologies, sources, and strategies, it seems unlikely that nation states will divest in these sensitive operational programmes as part of an initiative for international collaboration.

Furthermore there is no precedent, either practical or legal, as to how modern SIGINT technology could be used to target health risks in other countries. Detailed information on SIGINT is problematic to access, and it is therefore difficult to quantify the thresholds of defined threat that an infectious disease or PHEIC would have to meet to legally and ethically legitimate its use. The practical outputs and uses of SIGINT are therefore unclear as there is an inherent Catch 22 in its deployment. To anticipate an outbreak of an infectious disease, such monitoring would have to be done continuously to gather the data in a timely fashion; however, without the justification of an extant pandemic it is unlikely that an infectious disease will meet legal thresholds. In the case that a pandemic has already been declared, just cause is more likely, but comes too late. Furthermore, both public health surveillance and nation state surveillance should be focused on outcomes or objectives: without a clear goal there is no justification for mass data collection.

Finally, SIGINT is extremely costly in terms of resources, and it is questionable whether the value added to disease surveillance would indeed render it cost-effective. Due to the volume of information encompassed within SIGINT, significant human resources are required to assist in making SIGINT data into actionable intelligence (Aid 2003). GCHQ’s current programme requires over 300 analysts to filter through the data, and this cost can only increase, as the volume of electronic communications continues to expand (MacAskill et al. 2013). With competing nation-state priorities requiring the use of SIGINT, its application within global health may not be considered enough of a significant ‘threat’. As with OSINT, it may also bias a global north that is more technically capable.

There exists here a tension between the domains of intelligence and global health since both fields are governed by alternative paradigms that weigh human and security priorities by differing scales. The field of global health, motivated by equity, transparency, and collaboration, may find the employment of mass covert surveillance and dissemination between competing nation states an unacceptable tradeoff in the search for productive disease data (Koplan et al. 2009). It is clear that the dominating concept of ‘threats’ as they relate to disease entities is an unresolved debate within the field of global health and certainly between intelligence services and global health professionals (Bowsher et al. 2016). The global health security agenda has gained traction over recent years in the unfolding of SARS, Ebola, and Zika, yet this trend has received criticism also for signalling a departure from human-centred ethics towards a potentially discriminatory system prioritising the strategic priorities of the richest nations within the global health arena (Kickbusch et al. 2015). At the very least it is evident that ‘security’ is not a synonymous term within these communities, nor that it is clearly in either domain’s best operational interests to more closely align their respective meanings.

## Conclusion

It is clear that intelligence tools certainly could play a significant role in infectious disease outbreaks that are thought to threaten national and international security, through both public reporting and organisational analysis. The complex issue of when and how to utilise ‘clandestine’ national intelligence capabilities for global public good must now be a serious question given that these technologies exist and are already being put to use. The WHO has already deployed OSINT successfully and it seems likely that these tools will only expand in usage and sensitivity. Intelligence tools are not, however, sufficient on their own and can be used only to detect the presence of disease “chatter” and surrounding dynamics, while laboratory verification is needed to diagnose disease as it occurs in situ and health professionals to tackle the human and population components of care as outbreaks unfold.

Many questions are suggested from our analysis. First, the questions surrounding Civil-Military (CIMIC) cooperation cannot be avoided. Much of the technological and analytical expertise and equipment needed are those only used by the military and national intelligence services, while clinical expertise is almost exclusively cemented in international and national health systems. CIMIC is currently hotly debated, and such issues are certain to resurface here. For example, should epidemiologists become part of intelligence services? Do intelligence services outsource some of their technologies to medical specialists? These are crucial questions that must be addressed in future research. Furthermore, how would this work both structurally and legally? To what extent do infectious diseases meet the threshold of national security needed for them to sequester military and intelligence service resources and techniques and to what extent would this put the global health community at odds with advocates of human rights? It may be that intelligence tools offer only a substitute for alternative tools of health surveillance that avoid the ethical and practical dilemmas provided by covert bulk data collection. One such option employed in outbreak scenarios has been the use of anthropological methods and professionals within global health organisations based on the ground, mapping outbreaks in real-time (Ebola Response Anthropology Platform). Of course such programmes operate with their own resource constraints and limitations, but they serve to demonstrate that growing intelligence capabilities offer only part of a diversity of disease surveillance options.

Finally, since most infectious disease outbreaks are global, or transnational, they are therefore to a certain extent outside of a national jurisdiction. The question of governance remains at the forefront of concerns. Should extant intelligence technologies be divested to WHO? Do such operations fall within the remit of a foreign intelligence service of a high-income country, and if so, what accountability measures would be required? All of these questions are crucial to this discussion. We have provided an exploratory analysis of some intelligence techniques and how they could be applied to disease detection and reporting. It is hoped that this can provide the foundation for further in-depth study and debate into this under-researched, sensitive, yet enormously important field in global health.

## Footnotes

1 Anon. Interview with Technical Officer, Department of Global Capacities Alert and Response, World Health Organisation, (28 May 2015)
